# Measuring Muscle Mass and Strength in Obesity: a Review of Various Methods

**DOI:** 10.1007/s11695-020-05082-2

**Published:** 2020-11-06

**Authors:** Dionne Sizoo, Loek J. M. de Heide, Marloes Emous, Tim van Zutphen, Gerjan Navis, André P. van Beek

**Affiliations:** 1 Department of Health and Food, Campus Fryslân, University of Groningen, Leeuwarden, the Netherlands; 2 Center Obesity Northern Netherlands (CON), Department of Surgery, Medical Center Leeuwarden, Leeuwarden, the Netherlands; 3 Department of Internal Medicine, Division of Nephrolog, University Medical Center Groningen, University of Groningen, Groningen, the Netherlands; 4 Department of Endocrinology, University Medical Center Groningen, University of Groningen, Groningen, the Netherlands

**Keywords:** Obesity, Muscle mass, Methods, Body composition

## Abstract

Lower muscle mass in populations with obesity is associated obesity-related diseases like hypertension and type 2 diabetes mellitus. Bariatric surgery leads to sustained weight loss. During the weight reduction, loss of muscle should be minimized. Thus reliable quantification of muscle mass is much needed and therefore the also the need for validated methods. Imaging methods, magnetic resonance imaging and computed tomography scan, have been the gold standard for many years. However, these methods are costly and have limitations such as the maximum weight. Dual-energy X-ray absorptiometry is currently the most used alternative. Other, less expensive methods are very limited in their validation in populations with morbid obesity. This narrative review summarizes the current knowledge regarding measuring muscle mass and strength in obesity.

## Background/Introduction

Bariatric surgery is able to achieve sustained significant weight loss providing major health benefits [1, 2]. The focus of bariatric surgery is on reduction of fat mass (FM), as FM is an important determinant of co-morbidity, e.g., type 2 diabetes (TD2) and hypertension [3, 4]. However, muscle mass is equally important. The health risks of low muscle mass have been well documented in populations of older adults [5, 6] but awareness of low muscle mass and its health risks in obesity are lagging behind. In obesity, low muscle mass and function are associated with lower psychological health, quality of life and increased all-cause mortality [7, 8]. Furthermore, TD2 and hypertension are more common with lower muscle mass [9]. Accordingly, preservation of muscle mass during weight loss after bariatric surgery is clinically relevant. A recent study showed that after gastric sleeve surgery patients lost an average 10% lean body mass in the first month and 17% loss after 1 year [10, 11]. In preparing for surgery, patients on a very-low caloric diet lost more lean mass than FM [12]. These data underscore the need to decrease the loss of muscle mass both before and after the surgery. This implicates the need for validated methods to estimate muscle mass, which have already been established in healthy and elderly populations [13–15]. Validated methods for measuring muscle strength in populations with obesity might also be helpful, as muscle strength is positively related to muscle mass [16] and quality of life [17, 18]. In this paper, we review the methods of quantifying muscle mass and muscle strength, and their validation in populations with obesity.

## Methods

**Fig. 1:**
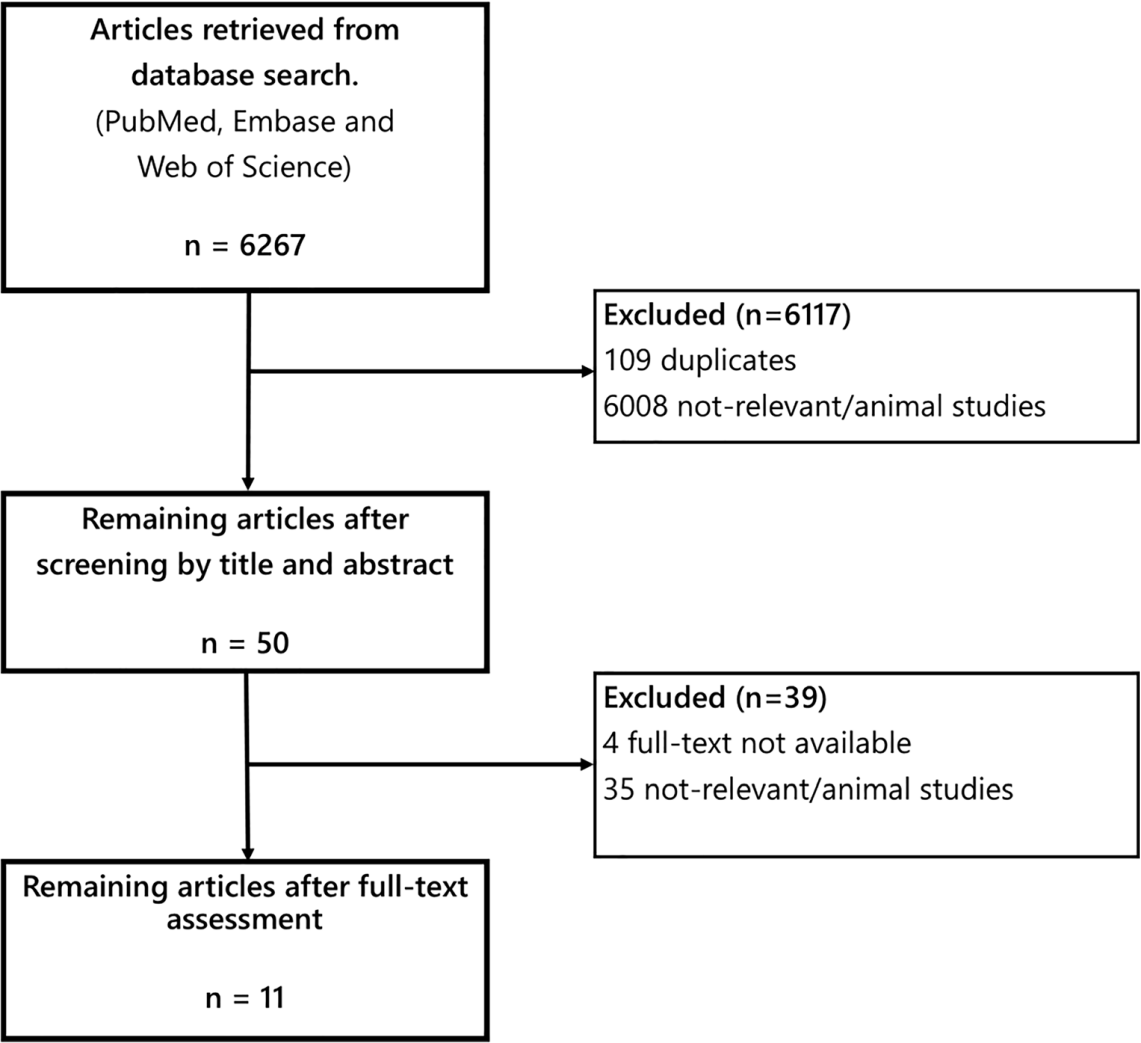
Flow-chart of systematic search

In July 2019, the review started with a systematic literature search on validating methods to measure muscle mass in populations with obesity (Fig. 1). The search was performed in Pubmed, Embase, and Web of Science. Search terms used were: “muscle*, lean mass, lean body mass, fat free body weight, muscular mass or lean body weight” and “obesit*, obese or adipos* and (“dual energy x ray absorptiometry, dexa, dxa, densiometr*” or “MRI, magnetic resonance imaging” or computed tomography, CT, tomography”). Selection was performed independently by two reviewers and consensus was obtained on the results. Of the 6267 articles found, 6117 were excluded based on title and abstract. After assessing the full test of the remaining 50 articles, 11 articles remained (Table 1). The articles finally selected contained mostly small numbers of subjects, usually less than 100, were very heterogeneous in characteristics of the population, gold standards and methods of measurement. We therefore concluded that a narrative review would be more appropriate for this topic. Articles found with the systematic search are included in the narrative review.

**Table 1:** Results of a systematic search into methods to measure muscle mass validated in populations with obesity

Author	Year	Population	Subjects with obesity	BMI	Gold standard	Comparison	Conclusion
Baker, S.T. [19]	2012	49 subjects	49	37.9 ± 0.9	DXA	MRI (single abdominal axial slice)	A single axial slice acquired through MRI in patients with obesity was a surrogate of total body skeletal muscle mass, measured by DXA.
Blue, M.N.M [20]	2018	61 subjects	61 (BMI ≥ 25)	33.5 ± 4.9	four-compartment model	DXA	The total error and standard error of the estimate results supported the use of DXA-derived measures of body volume, for estimates of body composition in individuals suffering from overweight/obesity using a 4C-model.
Boneva-Asiova, Z. [21]	2007	283 subjects	167	< 30	DXA	BIA (leg-to-leg)	The leg-to-leg BIA accurately assessed FM and FFM in a heterogeneous group of both sexes compared with DXA. Leg-to-Leg impendance lead to wide individual error in people with obesity and was therefore of limited value.
Bredella, M.A. [22]	2010	91 women	34	34.1 ± 4.7	CT (L3 and mid-thigh)	DXA	DXA was significantly inaccurate when used at the higher end of the weight spectrum in premenopausal women
Bucaloiu, I.D [23]	2011	70 women	70 (BMI ≥ 40)	48.3 ± 4.8	DXA	Alternative body size description	The FFM equation of Garrow et al. provided the best estimation of the DXA-measured FFM.
Jebb, S.A. [24]	2007	58 women	58 (BMI ≥ 25)	31.6 ± 2.5	Multi-compartment model	BIA (leg-to-leg)	The leg-to-leg system showed a good agreement with a three-compartment model.
Levitt, D.G. [25]	2010	29 women	29	46.7	Total body water	DXA (half-DXA)	Most values of the half-body DXA were accurately measured; however, bone and FM results were probably underestimated in individuals with obesity.
Savastano, S [26]	2010	45 women	45	42.1 [34.5–48.7]	DXA	BIA	The utility of BIA for accurately determining body composition was limited; however, BIA method was useful as an alternative to DXA in a selected clinical setting when repeated comparisons of body composition are required
Shafer, K.J. [27]	2009	132 subjects	42	33.7 [30.1–39.3]	DXA	BIA (MF)	Segmental MF-BIA significantly overestimated body fatness among adults with obesity.
Silver, H.J. [28]	2013	12 women	12	34.3 [30–38.2]	DXA	MRI (3 Tesla fat-water)	In the women with obesity studied, fat-water MRI had excellent concordance with DXA for the measurement of gross body lean soft tissue.
Ward, L.C. [29]	2007	157 subjects	45	[17.8–41.7]	DXA	BIA (MF and BIS)	MF-BIA data was accurate and mixture theory-based prediction scan be made; however, this technique is not yet universally applicable to all subject groups and BMI-adjustment has been recommended.

*BIA*, bioelectrical impedance analysis; *BIS*, bioimpedance spectrometry; *BMI*, body mass index; *CT*, computed tomography; *DXA*, dual-energy X-ray absorptiometry; *FFM*, fat-free mass; *FM*, fat mass; *L3*, third lumbar; *MF*, multi-frequency; *MRI*, magnetic resonance imaging

## Results

The results are divided into three topics: definitions of muscle mass, methods to measure muscle mass and measurement of muscle strength.

### Definitions of Muscle Mass

Many different terms are used in studies about muscle mass, e.g., lean mass or lean tissue mass, fat-free mass (FFM) and muscle mass. The muscle mass contains the weight of the muscles, including skeletal and smooth muscles. The term appendicular skeletal muscle mass only includes the skeletal muscles from the limbs [30]. The term lean tissue mass and FFM are not interchangeable. The lean tissue mass is the FFM together with essential fat (e.g., in bone marrow or in organs). This amount of essential fat is in non-obese individuals approximately 2–17% of the total FFM [31]. In adults, changes in lean tissue mass or FFM are generally due to changes in muscle mass, as the other components are known to be quite constant.

## Methods of Measuring Muscle Mass

### Imaging Methods

#### Magnetic Resonance Imaging

MRI has replaced the computed tomography (CT) scan as the gold standard [32]. MRI creates images from emission of radiofrequency energy from the nuclei of hydrogen atoms generated by magnetic fields and the signal will differ depending on the type of tissue [32]. MRI protocols can be optimized to assess the contrast among muscle tissue and fat tissue [33]. It also allows for quantification of FFM components, e.g., skeletal muscle mass, specific organ mass and bone marrow adipose tissue [14, 32]. MRI can also separate the muscle tissue into two different components, muscle with intramuscular fat and fat-free muscle. New methods to separate extracellular and intracellular muscle compartments can be used to show the expansion of extracellular space in aging and development of sarcopenia [34]. MRI is the most valuable tool for clinical research studies, due to the ability to quantify body compartments, which cannot be measured by other techniques. Furthermore, it can be used to assess relatively small changes over time which is useful in both intervention and observational studies [15]. Additionally, MRI can be used for body composition profiling, which is mainly used to compartmentalize the fat tissue but also to assess the weight muscle ratio [35]. General limitations of MRI are the costs, need for technicians, space requirements and infeasibility for patients with claustrophobia or with MRI incompatible implanted devices (e.g., cardiac pacemaker) [14]. The main drawbacks in a population with class II/III obesity are weight limitation (approx. 200 kg) and radial size limit (approx. 60 cm) [36]. MRI has been validated in healthy populations by post mortem cadaver studies in which subjects with obesity were also included, but with a maximum body mass index (BMI) of 31 kg/m^2^ (*r* = 0.97; Table 2). The results of test-retest and inter-observer reliability were highly correlated (respectively: 2.9%, *r* = 0.99 and 2.6%, *r* = 0.99) [33, 38]. There are MRI machines available with open configuration, eliminating the radial size limitations, and with higher maximal weight (approx. 290 kg) [39]. With these MRI machines, it might be possible to validate measurements of muscle mass in patients with morbid obesity. Finally, MRI can measure a single abdominal cross-sectional slice at the third lumbar vertebra (L3) level, which has been validated in individuals with obesity against total MRI imaging of the abdomen and allows for estimation of both visceral adiposity and skeletal muscle mass [19].

**Table 2:** Methods of measurement with their technical errors, advantages, disadvantages, and obesity specific disadvantages

Method of measurement	Validity*	Advantage	Disadvantage	Obesity specific disadvantage
Imaging methods				
MRI	< 1%	No radiation, specific quantification of body compartments	Costs, need for technicians, space requirements, infeasible for patients with claustrophobia or MRI incompatible implanted devices.	Weight limitation (approx. 200 kg), radial size limitation (approx. 60 cm)
CT	< 1%	Specific quantification of body compartments	Radiation exposure, costs, need for technicians.	Weight limitation (approx. 230 kg), radial size limitation (approx. 60 cm)
US	~ 2%	Non-invasive, fast, inexpensive, portable	Indirect quantification of muscle	Not validated in obesity
DXA	1–3%	Relatively cheap (compared to MRI and CT), high correlations to MRI and CT	Radiation exposure (although quite low), interference from dehydration and edema	Weight limitation (approx. 204 kg), scanning area (approx. 60 cm)
Anthropological methods				
Circumference	-	Non-invasive, fast, portable, inexpensive	Indirect method, no distinction between FFM and FM	Difficulties in measurement due to excess fat
SFT	3–5%	Fast, portable, inexpensive	Indirect method	Less precise in populations with obesity
Other methods				
BIA	< 2%	Non-invasive, fast, inexpensive	Indirect quantification of muscle, many factors influence outcome	Underestimation of total body fat and overestimation of muscle mass
ADP	1%	Non-invasive, fast	Expensive, indirect quantification of muscle	Underestimation of total body fat and overestimation of muscle mass

*Validity according to Mersebach et al. [37]

*ADP*, air displacement plethysmography; *BIA*, bioelectrical impedance analysis; *BMI*, body mass index; *cm*, centimeter; *CT*, computed tomography; *DXA*, dual-energy X-ray absorptiometry; *FFM*, fat-free mass; *FM*, fat mass; *kg*, kilograms; *MRI*, magnetic resonance imaging; *SFT*, skinfold thickness; *US*, ultrasound

#### Computed Tomography Scan

In 1971, the CT scan was the first clinically accepted body composition measurement tool and was used as a gold standard [34]. The CT scan uses an X-ray beam to make cross-sectional images of the body, which can estimate total body fat, visceral fat and skeletal muscle mass [40, 41]. A single cross-sectional image of the abdominal area at the level of the L3 has shown a high correlation with total body skeletal muscle mass and body adipose tissue, comparable to whole-body MRI results [42]. However, these studies have mainly been done in healthy populations and populations prone to wasting and thus might not be applicable in patients with obesity [43]. General limitations of CT are the costs, need for technicians and radiation exposure [14]. Obesity-related limitations are similar to MRI, as CT also has a weight limit (approx. 230 kg) and radial size limit (approx. 60 cm) [15]. The CT validity was also tested with cadaver dissection in non-obese individuals (*r* = 0.97; Table 2) [33]. The test-retest reliability showed a high correlation (1.4%, *r* = 0.999) [33, 44]. Currently, CT is rarely used to measure muscle mass.

#### Dual-Energy X-ray Absorptiometry

DXA is the best alternative to the gold standard. Originally invented to assess bone mineral content, DXA is now frequently used to examine body composition and muscle mass. It uses two X-ray beams to separate fat, bone and lean tissue based on the tissue X-ray absorption [45]. DXA highly correlates to both MRI and CT measures of skeletal muscle mass [22, 46, 47]. The main limitation is that muscle is not quantified directly. Several assumptions are made to calculate the lean soft tissue mass or FFM, based on the different gray-tones in the DXA scan. There can also be interference from both dehydration and edema, which are not unusual in populations with obesity [48, 49]. The validity of the muscle measurements of the DXA compared to CT has been examined in non-obese volunteers (*r* = 0.96; Table 2) [50]. The test-retest reliability between measures of FFM, also showed a high correlation (*r* = 0.99) [47]. However, these studies excluded individuals who did not fit within the DXA field-of-view or exceeded the CT radial size [50]. A recent study has shown that half-body analysis is comparable to the whole-body analysis for FM, non-bone lean mass and fat percentage. This half-body analysis uses a symmetry assumption, in which the left part of the body should have the same fat and muscle distribution as the right part of the body. It can be used if patients do not fit in the DXA field-of-view [51, 52]. In short, the DXA can accurately predict the FFM, in both healthy individuals and individuals with obesity.

#### Ultrasonography

In ultrasonography (US), high-frequency sound waves travel through the skin and are reflected by the underlying tissues. The reflection is called the acoustic impedance and each tissue type has a unique impedance. The reflection, received by the transducer, is converted into electrical signals and then visualized on a monitor [53]. The ultrasound is a non-invasive, fast and inexpensive tool [53, 54]. Alterations in the muscle mass can also be measured with US [55]. The ultrasound is portable and therefore especially useful in critically ill patients at bedside [53]. There have been studies in healthy and older adults validating US in quantifying body composition, e.g., FM, especially visceral fat, and skeletal muscle thickness [55–57]. FM measurements with US have been validated with DXA in non-obese individuals (*r* = 0.98; Table 2) [58]. Estimation of lean tissue mass with US has not been validated in populations with obesity. The most commonly used sonographic devices have a maximum measuring depth of approximately 12 cm [59]. This limits the use in persons with morbid obesity, in whom subcutaneous fat thickness of more than 12 cm is not uncommon. Using an ultrasound with a lower frequency can solve this problem, as the penetration increases, however this will decrease the resolution of the image [60]. Future research on US to measure lean tissue in populations with obesity is underway. Finally, it might also be possible to look at muscle quality, which has already been validated in healthy athletes and critically ill patients [61, 62].

#### Summary of Imaging Methods of Measuring Muscle Mass in Obesity

MRI and CT have been the gold standard in measuring muscle mass for years. Both have a weight and radial size limit. Newer models have bigger radial size limits and there is even an open configuration MRI [34]. However, both MRI and CT are expensive and require trained personnel. Currently, the CT is rarely used for measuring muscle mass.

The DXA results (lean tissue mass and FFM) highly correlate with both MRI and CT measures of muscle mass and DXA is relatively cheap compared to MRI or CT [63]. DXA also has some limitations, e.g., need for trained personnel and (low levels of) radiation. DXA is already established and frequently used as an alternative to MRI to measure muscle mass. US to measure muscle is quite new and has not been tested in populations with obesity yet.

### Bioelectrical Impedance Analysis

Bioelectrical impedance analysis (BIA) measures the electrical impedance (or flow) of an electric current through the body. Due to differences in electrical conductance, the total body water, FFM and body fat can be estimated [48]. The FFM is estimated based on a constant hydration of FFM of 73%. The hydration of the FFM is higher in obesity (approx. 77.5%) [48, 64]. Consequently, most prediction equations give an underestimation of the FM and an overestimation of the FFM when used in participants with obesity [65]. BIA has been validated against MRI in healthy subjects, but has not been done in a population with obesity (*r* = 0.87; Table 2) [66]. BIA is unreliable when compared to DXA results in a population with morbid obesity, overestimating the FFM with increasing errors as the BMI increases [21, 26, 27, 29, 48].

Furthermore, factors that can influence the outcome of the BIA measurement are food, alcohol, physical exercise, time of day, skin condition (perspiration), body shape, and some disease conditions or treatments [67].

### Air Displacement Plethysmography

Air displacement plethysmography (ADP) is currently mostly performed in a BOD POD (Bod Pod Body Composition System, Concord, CA, USA), which calculates body volume. The difference in volume of the chamber, with versus without the patient, is the total body volume. The body volume can then be used to estimate the percentage of body fat [68, 69]. The relative body fat is calculated using the following equation: percent fat = 495/density − 450 [68, 70]. Similar to the BIA, the ADP uses assumptions for calculating the FFM. This can give an underestimation of the FM and an overestimation of the FFM in persons with obesity [14]. ADP has been compared to hydrostatic weighing for its validity in measuring percentage body fat (*r* = 0.93; Table 2) [68]. Hydrostatic weighing also uses total body volume together with the same assumptions to calculate FM and FFM [71]. A validation study with ADP against DXA showed an overestimation of body fat in participants with a lower BMI and an underestimation of body fat in participants with a high BMI [72].

### Anthropometric Methods

#### Circumference and Skinfold Thickness

Circumference measurements are done with a flexible quilting tape, making the measurements safe, easy and at low-cost [73]. The accuracy of circumference measurements depends on the skills and training of the person taking the measurements and can differ between observers [74]. Arm, thigh, and calf circumference together with skinfold thickness (SFT) can be used to estimate skeletal muscle mass. A prediction equation was developed to estimate the skeletal muscle mass: skeletal muscle mass = height × [0.00744 × corrected arm girth^2^ + 0.00088 × corrected thigh girth^2^ + 0.0041 x corrected calf girth^2^] + 2.4 × sex − 0.048 x age + race × 7.8. The correction of the circumferences is done by subtracting π x SFT from the measured circumference [75]. The equation was validated with MRI in patients with class I and II obesity [76].

### Biochemical Measurements

#### Creatinine

Creatinine is a breakdown product of creatinine phosphate and is released from muscle cells with a usually fairly constant rate in to the blood. The classic method for the estimation of total skeletal muscle mass is 24-h urinary creatinine excretion, with the equation of 17–20 kg of muscle mass per gram of creatinine [77, 78]. This equation has been validated in patients with advanced renal failure, children, adolescents, elderly people and patients with wasting conditions, but not in populations with obesity [78–80]. One study also highlighted the relatively high day-to-day variability of creatinine excretion (approx. 4–8%) and some influential factors on the secretion of creatinine (e.g., diet, exercise, menstrual cycle). It shows a high validity compared to anthropometric methods [78]. Serum creatinine can estimate muscle mass when serum cystatin C is added to the equation.^50^ The equation has been developed and validated in a diverse population, only excluding patients with chronic kidney disease (*r* = 0.93) [81]. However, it has not been specifically validated in populations with obesity. Another relatively new tool is the D_3_-creatine dilution method. This method measures D_3_-creatine and D_3_-creatinine excretion in morning urine [82]. Creatine is needed for energy supply in the muscle and approximately 2% is converted into creatinine and excreted. With the D_3_-creatine method, subjects will ingest a single dose of stable isotope-labeled D_3_-creatine. D_3_-creatine will be taken up into muscle tissue and the excreted D_3_-creatinine will give an estimation of skeletal muscle creatine enrichment and thus total body creatine and total muscular mass [82–84]. There is less variability compared to 24-h urinary creatinine excretion and less dependence on subject compliance [83]. This specific method of measuring D_3_-creatine has yet to be validated in a population with obesity.

## Muscle Strength

### Isokinetic Testing

Isokinetic testing is the gold standard method to measure muscle strength. There are two main methods to measure isokinetic strength. The first method is the peak torque, which is the maximum amount of force that can be used to extend or flex the muscles around the knee [85, 86]. Both strength and speed can be controlled during the measurement, leading to a more detailed assessment [87]. Studies in healthy men and elderly populations have found a correlation between isokinetic torque values and muscle mass and total muscle strength [88–90]. The second method is the one repetition maximum, in which the maximum weight that can be pushed once is measured [86]. It is usually measured with extension measures with a weight and pulley system. This method is less appropriate for vulnerable groups (e.g., elderly, cardiac and hypertensive patients), due to the chance of over bearing [91]. Neither one of these two methods has been studied in patients with obesity.

### Grip Strength

A simple method to evaluate muscle strength is the hand-dynamometer, which measures the grip strength. Compared to isokinetic testing, the grip strength has shown to be a good alternative for measuring muscle strength [92]. It is associated with total body strength and total body muscle mass [93, 94]. Studies in assisted-living and community-dwelling older adults have shown that grip strength is associated with muscle mass and can help predict the FFM index (kg FFM/length) [5, 6]. These studies have only been performed in sarcopenia prone populations, without including individuals with obesity. Studies in populations with obesity mostly look at the measurement of physical fitness and are not related to muscle mass [95].

## Discussion

MRI has replaced the CT scan as the gold standard for measurement of body composition, and the DXA scan is a good alternative. Limitations of these measurements are not uncommon when evaluating muscle mass in obesity. Most limitations regard to weight per se or difference in body composition compared to non-obese subjects and dealing with these limitations should be part of methodology. Unless the weight threshold is identified prior to the patient attending, there is potential for people with obesity to face a stigmatized situation in a routine clinical encounter. This needs to be avoided, with a clear need to make these diagnostic procedures more accessible to people with obesity. Furthermore, only a few methods have been validated in populations with obesity, especially in the higher classes of obesity. Further research should look at different methods to measure muscle mass in obesity, to find cheaper methods with less limitations regarding weight and size which can be made widely available for clinical practice.

## Abbreviations

ADP: Air displacement plethysmography
BIA: Bioelectrical impedance analysis
BMI: Body mass index
CT: Computed tomography
DXA: Dual-energy X-ray absorptiometry
FFM: Fat-free mass
FM: Fat mass
L3: Third lumbar
MF: Multi-frequency
MRI: Magnetic resonance imaging
SFT: Skinfold thickness
TD2: Type 2 diabetes mellitus
US: Ultrasonography
